# Disentangling the Impacts of PAHs, Microplastics, and Sediment Resuspension on Algal Physiology: A Partial Least Squares Structural Equation Modeling Approach

**DOI:** 10.1021/acsenvironau.5c00060

**Published:** 2025-07-23

**Authors:** Hoi Shing Lo, Betty Chaumet, Alyssa Azaroff, Anna Sobek, Sofi Jonsson, Elena Gorokhova

**Affiliations:** Department of Environmental Science, 7675 Stockholm University, Stockholm SE-106 91, Sweden

**Keywords:** polycyclic aromatic hydrocarbons, microplastics, sediment resuspension, algal physiology, PLS-SEM

## Abstract

Environmental stressors, such as contaminants and physical factors, rarely act in isolation, and studying their joint effects provides a more accurate reflection of real-world scenarios. To capture these interactions and disentangle the direct and indirect influences on algal responses, we applied partial least squares structural equation modeling (PLS-SEM), allowing us to reveal the hierarchical relationships among stressors and their cumulative impact on algal physiology. We examined combined effects of microplastics (MP; presence/absence), polycyclic aromatic hydrocarbons (PAHs; a mixture of acenaphthene, fluorene, phenanthrene, and fluoranthene at a total chemical activity in the sediment of 0 or 0.14), and sediment resuspension (turbidity: 0.8–3.9 NTU) on *Ceramium tenuicorne*, a coastal macroalga that is likely to encounter all these stressors in its natural habitats. Mechanical mixing at two intensities (low and high) was applied as an experimental treatment to induce resuspension. The analysis separated the effects of mechanical mixing and turbidity, given their nonlinear relationship, as stronger mechanical mixing did not consistently result in proportional turbidity increases. The algal physiological responses were evaluated using changes in pigment composition (Chl *a*, Chl *c*, and carotenoids), photosystem II (PSII) performance, total antioxidant capacity, and algal stoichiometry measured as elemental (%C, %N, %H, and C/N) ratios. We found that PAH exposure was the main suppressor of pigment concentrations and PSII performance, underscoring the mechanisms of its adverse effects on the photosynthetic machinery and nutrient assimilation. Moreover, stronger turbulence further decreased pigment concentrations, while sediment resuspension increased antioxidant capacity in algae, possibly due to physical damage from abrasion and scouring. We also found that MP addition significantly increased turbidity, thus aggravating the effects of the sediment resuspension. In conclusion, we provide a mechanistic explanation of how the combined exposure to MPs, PAHs, and sediment resuspension can impact pigment composition, photosynthesis, and stoichiometry of the algae, leading to decreased productivity.

## Graphical abstract

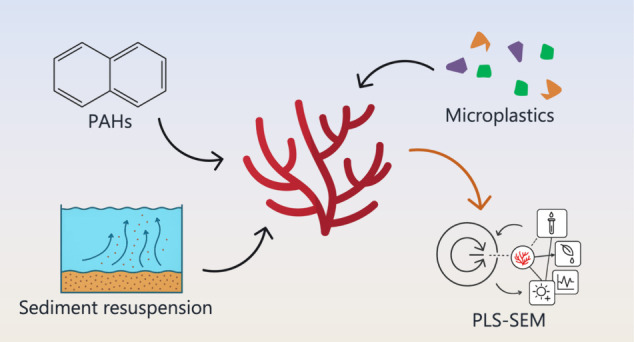

## 1 Introduction

Environmental contaminants and other stressors rarely act in isolation, and their relationships are usually complex.^1^ In addition to the mixture effects, the behavior and impact of pollutants are influenced by nonchemical environments. For example, sediment-bound hydrophobic organic contaminants (HOCs), such as polycyclic aromatic hydrocarbons (PAHs), are more actively released into bottom water during resuspension events.^2^ When these events occur frequently, they can significantly influence the release and mobility of contaminants,^3^ which is often overlooked in conventional risk assessment frameworks despite their acknowledged ecological and environmental impact.^4^ Thus, understanding the interactions between contaminants and suspended solids is essential for accurately forecasting the ecological impacts of sediment resuspension. Therefore, studying the combined effects in experimental settings provides a realistic simulation of environmental dynamics, enabling assessment of various factor combinations.

HOCs tend to accumulate in sediments because of their low water solubility and strong affinity for organic matter, which promotes their long-term storage in calm, low-energy environments such as lentic waters. This is why, under low disturbance, sediments are usually a sink for hydrophobic pollutants.^5^ In contrast, particles become less stable in more hydrologically dynamic environments with significant biological activities (e.g., bioturbation), strong currents, or physical disturbance (e.g., systems undergoing dredging operations^6^). These activities lead to sediment resuspension when water movement generates shear stress that exceeds the critical threshold required to mobilize sediment particles. This threshold depends on the sediment properties, including particle size, density, and the cohesiveness of the matrix.^7^ Once in the water column, these contaminants can disperse and become bioavailable.

Microplastics (MPs, <1 mm), which are particles generated by the fragmentation of plastic litter, pose environmental concerns due to their widespread presence and ability to influence sediment and contaminant behavior. High MP loads were observed in numerous aquatic habitats subjected to frequent resuspension, such as coastal zones,^8^ coral reefs,^9^ and oceanic trenches,^10–13^ coinciding with various contaminants, including HOCs.^14^ As these chemicals adhere to the hydrophobic surfaces of particulates, including plastics, forming complex contaminant mixtures.^15^ Often MPs have a lower specific gravity than mineral particles (i.e., clay, silt and sand), making them more likely to be resuspended and remain longer in the water column.^16^ Although sorption capacities of MPs are similar between HOCs and sediment organic carbon, their greater tendency to resuspend may facilitate the dispersion of both MPs and associated contaminants in the water column, increasing their potential to create localized pollution hotspots.^16^ Yet, the extent to which MPs influence contaminant release, transport, and bioavailability during resuspension events remains poorly understood.

Designing experiments to study the effects of combined stressors remains challenging due to diverse exposure pathways, variability in species sensitivity, difficulty selecting ecotoxicologically relevant test organisms, and the need to balance ecological relevance with feasibility.^17^ Coastal macroalgae emerge as suitable test organisms because they play foundational roles in aquatic ecosystems, and are relatively easy to maintain in laboratory. In their natural habitats, they are frequently exposed to combined stressors, e.g., plastic litter,^18^ terrestrial runoff,^19^ and sediment resuspension,^20^ making them highly relevant target organisms for assessing their joint impacts.

When coastal macroalgae to access combined stressors, however, conventional single-end point tests often fail short, as they cannot disentangle the interactions between chemical and physical drivers. To address this, multiend point, hierarchical approaches are needed or methods that capture both direct and indirect responses, supporting more ecologically meaningful evaluation. In this context, *hierarchical effects* refer to causal chains where a stressor first affects a proximal cellular or biochemical mediator (e.g., pigment concentration), which in turn influences a distal physiological end point. In addition, stressors interaction, in which one stressor modifies another, with the altered stressor then affecting physiological end points, must be considered to understand how complex, co-occurring drivers propagate through biological levels and ultimately determine organismal performance in real coastal environments.

The aim of this study was to identify and quantify the direct and indirect effects of PAHs, MPs, and sediment resuspension on the physiology of the coastal macroalga *Ceramium tenuicorne* using a multiend point experimental design and partial least-squares structural equation modeling (PLS-SEM). PAHs were selected as model substances because they are widespread, persistent contaminants with well-characterized toxicological profiles. In particular, PAHs are harmful to plants, impairing photosynthesis and inducing oxidative stress,^21^ which makes them ideal for investigating mechanistic effects on algal physiology.

To achieve the aim, we investigated the physiological responses of *C. tenuicorne* to combined exposure to PAHs, MPs, and sediment resuspension driven by turbulence. The biological end points included pigment concentrations (chlorophyll [Chl *a*], Chl *c*, and total carotenoids), photosystem II (PSII) performance, total antioxidant capacity (assayed by Oxygen Radical Absorbance Capacity; ORAC), and the elemental stoichiometry (percentages of carbon [%C], hydrogen [%H], and nitrogen [%N] as well as C/N ratio), which together reflect key aspects of algal health and functionality. Using PLS-SEM, we examined both direct and indirect pathways linking these stressors to physiological algal responses. Specifically, we tested whether pigment loss and antioxidant capacity can forecast subsequent alterations in photosynthesis and thereby influence the elemental ratios of the algae. This approach enabled us to disentangle causal relationships and gain mechanistic insight into how combined stressors impact algal health and functionality under ecologically relevant conditions.

## 2 Materials and Methods

### 2.1 Experimental Design

The experiment was designed to mimic a coastal environment impacted by anthropogenic pollution and to investigate the individual and combined effects of PAHs and MPs on the marine macroalga *C. tenuicorne* under sediment resuspension. Artificial sediment was prepared to simulate Baltic Sea sediments in terms of grain size distribution and composition (Text S1 of Supporting Information). Four treatments were used: (1) sediment contaminated with PAHs, (2) sediment contaminated with MPs, (3) sediment contaminated with both PAHs and MPs, and (4) uncontaminated sediment (control). The test concentrations of MPs and PAHs were based on reports from heavily polluted areas (Table S1 of Supporting Information), and test sediments were prepared as described in Text S1 of Supporting Information. As environmentally relevant MPs, polyethylene (PE) was chosen because it is the most widely produced polymer globally.^22^ Although virgin PE is slightly less dense than brackish water, a thin biofilm, aggregation with other particulates, or incorporation into faecal pellets are sufficient to facilitate deposition to the seabed,^16^ and, as a result, PE is a ubiquitous MP in sediment and water.^23^ Thus, when preparing the test sediment, PE sheets (1 × 1 mm) were mixed with the artificial sediment at 0.4 mg kg^–1^ dw. The MP-conditioned sediment ensured that all treatments started with an identical MP pool, allowing the effects of resuspension to be isolated.

Each of these treatments was subjected to two sediment resuspension regimes, with two mixing intensities applied to generate different degrees of turbulence. This turbulence involves not only sediment resuspension, which specifically reflects the presence of suspended particles in the water, but also water movements that can independently impact algae. The relationship between turbulence and turbidity was not linear, as increased turbulence did not consistently lead to proportional changes in turbidity, representing conditions ranging from quasi-stagnant to highly turbid.^24^ Consequently, turbulence (low vs high) and turbidity (between 0.8 and 3.9 NTU) were treated as categorical and continuous variables, respectively.

#### 2.1.1 Algal Culture

The filamentous red alga *C. tenuicorne* is a common species in the Baltic Sea, inhabiting coastal areas with salinities from 2 to 30 PSU. These habitats can occasionally be turbid, which influences the algal distribution.^25^ The alga employed in this study is a strain derived from the Baltic Sea and was cultivated in natural, filtered, autoclaved water at a salinity of 6.8. The water was enriched with nutrients, vitamins and trace elements following the guidelines^26^ to support optimal growth. The female clones used in the experiment were grown in sterilized polystyrene Petri dishes at 22 ± 1 °C with a light intensity of 35 ± 5 μmol m^–2^·s^1^ and a light/dark regime of 14:10 h. Top branches were transferred to a new medium weekly to ensure a constant supply of actively growing algae.

#### 2.1.2 Chemical Activity

We used chemical activity to represent the PAHs dose in the exposure system and the algae. Chemical activity refers to a molecule’s potential to partition and react within a matrix (e.g., water), capturing its bioaccumulation and baseline toxicity (narcosis) potential.^27^ As a cumulative exposure metric, chemical activity expresses the effective concentration relative to solubility. This allows mixture effects to be treated as additive (at least for the baseline toxicity) and to consider only the freely dissolved bioavailable fraction. The chemical activity in the algae was calculated using eqs 1 and 2.

$$C_{\mathrm{free}}=C_{\mathrm{algae}}/K_{\mathrm{oc}}$$ 1

$$a=C_{\mathrm{free}}/S_{L}$$ 2

where *C*_free_ (in mg L^–1^) and *C*_algae_ (in mg kg^–1^ of lipid) are the PAH concentrations in water and algae, respectively, *K*_OC_ is the partition coefficient between organic carbon and water (L kg^–1^), and *S*_L_ is the subcooled liquid solubility (mg L^–1^) of the PAHs in water.^28^

The baseline toxicity is commonly observed in the chemical activity range of 0.01 to 0.1;^29^ however, specific effects related to photosynthesis biochemistry can also arise within the same dose range due to the reactive nature of PAHs, especially those with low molecular weight.^30^ The interaction of PAHs with the photosynthetic machinery may extend beyond baseline toxicity and include specific effects, such as interference with PSII, oxidative stress or damage to pigments.^31–33^ Thus, in the context of this study, the relevance of chemical activity lies in its ability to reflect the bioavailable fraction of PAHs that can interact with biological systems, regardless of the mode of action, and that it allows for summation of the total chemical activity of the mixture. By expressing dose in terms of chemical activity, the experiment provides a consistent and comparable measure of the bioavailable fraction of the PAH mixture,^29^ reflecting its potential to interact with biological membranes and disrupt photosynthetic processes, whether through baseline toxicity or specific mechanisms such as pigment degradation.^31^

#### 2.1.3 Exposure System

The prepared sediments (in triplicate for each treatment group; Table S2 of Supporting Information) were added to polycarbonate cylinders (30 cm tall; 4.6 cm internal diameter) to a height of 3 cm (∼100 g of sediments). Then, 400 mL of the artificial brackish water with salinity 7 ‰ and pH 8 (Text S2 of Supporting Information), corresponding to the culture medium of *C. tenuicorne*, was carefully added to each cylinder. A stir bar was placed 12 cm above the sediment surface. The system was allowed to stabilize at 21 °C for 9 days to achieve chemical equilibration between the sediment and the overlying water.

After the equilibration period, *C. tenuicorne* (∼120 mg wet weight) was placed 20 cm above the sediment surface using a metal net to hold it in place (Figure S5 of Supporting Information). This setup (1) prevented direct contact with the sediment, ensuring PAH uptake occurred only via resuspended particles, and (2) reflected the natural habitat, where the species typically forms loose, drifting mats a few decimeters above soft Baltic bottoms.^34^ The cylinders were mounted on a stand with a central rotating platform controlled by a motor to induce sediment resuspension at rotating speeds of 6 or 18 cm s^–1^ for low and high turbulence scenarios, respectively. The experiment lasted for 7 days, during which sediment resuspension was assessed by turbidity measurements (2100NIS, HACH, U.S.) on days 1 and 7. The 7-day exposure period was selected based on the standardized growth inhibition assay with *C. tenuicorne*, which ensures reliable and sensitive detection of biological responses over this duration.^35^ At the experiment termination, the algae were collected for analysis of photosynthesis performance, biomarkers, elemental ratios, and PAH concentration.

The methodologies of the analysis are described in detail in Text S3 of Supporting Information. Briefly, photosynthesis performance was probed using a DUAL-PAM-1000 portable chlorophyll fluorometer (Walz, Germany), following the protocol established by Burritt & MacKenzie^36^ for dark adapted samples. Maximum photochemical efficiency of PSII (Fv/Fm), which indicates the maximum efficiency of PSII when all reaction centers are open; (2) nonphotochemical quenching coefficient (NPQ) were obtained. Pigments (Chl and carotenoids), total antioxidant capacity, PAH and elemental analyses were performed using lyophilized algae.

### 2.2 Data Analysis

Statistical analyses were performed to evaluate the effects of experimental factors on biological responses using a combination of generalized linear models (GLMs) and partial least squares structural equation modeling (PLS-SEM). GLMs were employed to assess the overall impact of the experimental factors on the biological responses. These models were selected for their ability to accommodate non-normal distributions and account for potential interactions between the predictors. Further, potential hierarchical relationships and mediating effects among the experimental factors and the biological responses were explored by PLS-SEM. The PLS estimator does not assume multivariate normality^37^ and this method allows the construction and analysis of path models that depict causal relationships among variables based on the hypothesized connections, which is particularly useful for understanding complex systems where variables may influence each other in hierarchical or mediation pathways.

#### 2.2.1 Overall Effects of the Experimental Conditions on Algal Responses

GLMs with normal error structure and log-link or identity functions (Statistica 14.0.0.15, TIBCO Software Inc., US) were used to evaluate the effects of the experimental factors on variation in Chl (*a+c*), total carotenoids, relative Fv/Fm, Y­(II), NPQ, total antioxidant capacity, %C, %H, %N, and C/N. In all GLMs, MPs (categorical), chemical activity of PAHs in algae (continuous), and turbidity (continuous) and their two-way interactions were assigned as potential predictors. Nonsignificant interactions were omitted from the final models. The Wald statistic was used to evaluate whether the regression coefficients were statistically significant (*p* < 0.05). Model goodness of fit was checked using deviance, and Pearson χ2 statistics and model residual plots were assessed visually to exclude remaining unattributed structures indicative of a poor model fit. Chl (*a+c*), carotenoids, Y­(II), and ORAC were Box-Cox transformed to improve homogeneity and approach the normal distribution of the model residuals. The missing data on Fv/Fm, Y­(II) and NPQ (4 cases in total) were imputed using EM (maximum expectation likelihood) algorithm, which assumes a multinormal distribution model for the data^38^ with 1000 permutations as implemented in Primer 7 v.7.0.21 software.

Effect Size Index (RESI; denoted as *S*_β_) was used to assess the effect size of significant predictors on the following scale: small (<0.1); medium (0.1–0.25); and large (0.25–0.4) effects. RESI provides a robust alternative for effect size assessment in GLM because it addresses the challenges of interpreting effect sizes on the multiplicative scale. Unlike traditional effect size measures, which may not align well with the log-transformed response scale of these models, RESI provides an interpretable measure of relative change by expressing effects as a percentage change relative to a baseline or control.^39^ RESI can be transformed into other common effect size measures such as Cohen’s *d* (for binary predictors) and *f* ^2^ (for continuous predictors), allowing for the estimation of the minimum effect size required. *S*_β_ values were estimated for each significant predictor by comparing the full model (containing all predictors) to a reduced model (excluding the specific significant predictor), thus isolating the unique contribution of each significant predictor. The RESI estimation and the conversions of *S*_β_ to *f* ^2^ or Cohen’s *d*, were implemented using the RESI package^40^ in the R 4.1.1 environment via the functions resi_pe­(), S2 fsq­(), and S 2d­(). These effect size values were then used in G*Power 3.1.9.7 to determine the minimum required sample size in the GLMs.

#### 2.2.2 Specific Hypotheses and Structure for PLS-SEM

PLS-SEM was used to investigate the proximate drivers of the observed responses and mediation (i.e., indirect) effects among the stressors. The specific hypotheses (H_1_–H_6_) and their rationale are outlined in Table 1 and Figure 1.

**Figure 1:**
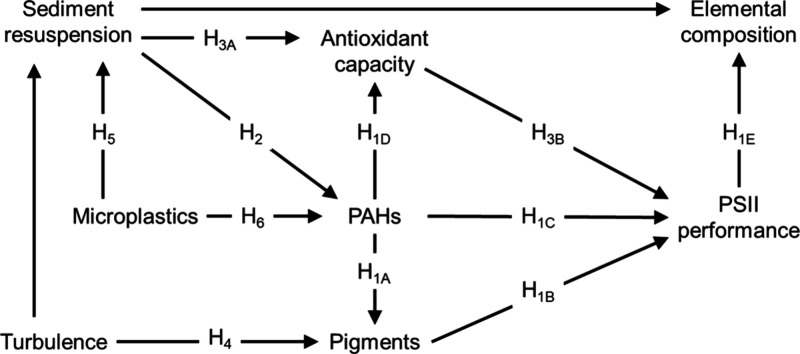
Conceptual pathways of interactions between stressors and biological responses and the proposed hypotheses (H); H_7_ and H_8_ are treated as indirect effects.

**Table 1:** Hypotheses (H) and Their Rationales in PLS-SEM

hypothesis	rationale
**PAH effects**	
**H**_**1**_ **:** PAH exposure has adverse effects on algal physiology through multiple and interlinked mechanisms.	**A:** Pigment reduction in algal cells (Asghari et al., 2020; Jajoo et al., 2014) that leads to **B:** Decreased PSII performance; **C:** Direct inhibition of PSII due to thylakoid membrane damage (Kreslavski et al., 2017); **D:** Increased antioxidant defenses triggered by ROS production due to exposure (Othman et al., 2023); **E:** Reduced photosynthetic efficiency, alters elemental composition (%C, %H, %N) (Ajdanian et al., 2020; Shamsipur and Pashabadi, 2018; Figueroa et al., 2003). Differential decreases in %C and %N would result in an altered C/N ratio.
**sediment resuspension effects**	
**H**_**2**_ **:** Resuspension increases PAH uptake by algae due to PAH release from sediment.	Sediment resuspension facilitates PAH release into the water, increasing the bioavailability.
**H**_**3**_ **:** Sediment particles exert shear stress on algae under high turbulence, affecting antioxidant capacity and PSII performance.	**A:** Shear stress from sediment particles causes mechanical wounding, facilitating antioxidant production in algae (Edwards, 1969; Suzuki and Mittler, 2012). **B:** The enhanced antioxidant capacity protects photosynthetic machinery, supporting PSII performance (Martinez et al., 2014) and counteracting PAH effects on photosynthesis.
**H**_**4**_ **:** Intensive mixing affects pigment concentrations in algae, with context-dependent outcomes.	**A:** Turbulence may increase pigment concentrations by reducing boundary layer thickness, enhancing CO_2_ diffusion and chlorophyll production (Estrada and Berdalet, 1998). **B:** Conversely, it may decrease pigments by damaging structure and function of photosynthetic appendages (Asaeda and Rashid, 2017).
**MP effects**	
**H**_**5**_: MPs increase water turbidity by destabilizing the sediment bed.	MP low specific gravity leads to prolonged suspension in water and higher turbidity (Hope et al., 2021).
**H**_**6**_ **:** MPs facilitate PAH accumulation in algae by prolonging exposure through sustained PAH release in the water column.	MPs, by remaining suspended in the water column, provide a prolonged source of PAHs, increasing the potential for algal uptake. Indirect effects through sediment resuspension are covered in H2.

The model was constructed to examine relationships among the latent variables (LVs, all reflective constructs; Table S4 of Supporting Information), each represented by one or several indicators and addressing one or more hypotheses. The outer (measurement) model was assessed using factor analysis and indicators with low loadings/weights (<0.5) and *p* > 0.05 were removed.^200^ Construct reliability and convergent validity for each LV were evaluated using the average variance extracted; AVE value >0.5 indicated that a minimum of 50% of the indicator variance was explained. Cronbach’s alpha and the reliability coefficients (Rho a and Rho c) were used to assess the descriptive power of the indicators toward each LV (cutoff value: 0.7).^200^ Standardized root mean squared residual (SRMR) was used to measure the overall model fit (cutoff value: 0.080).^41^ Finally, the heterotrait-monotrait ratio of correlations (HTMT, cutoff value: 0.9) was employed to assess discriminant validity between the constructs.^42^

The structural model established significant paths to address the hypotheses (Figure 1). We used power analysis (G*Power 3.1.9.7; Text S4 of Supporting Information) to estimate sufficient sample size for *R* ^2^ reliability for each path (fixed model, single regression coefficient). With specified paths, PLS bootstrapping (10,000 bootstraps) was utilized to compute path coefficients with *t* values and corresponding *p* values, as well as total indirect and specific indirect effects. The *R* ^2^ statistic explained the variance in endogenous variables and the effect size (*f* ^2^) along with the bias-corrected confidence interval (CI) were used to quantify the magnitude of the predictor’s contribution to the model and assess the practical significance of the predictors. To determine the practical relevance of significant effects, *f* ^2^ values ranging from 0.020–0.150, 0.150–0.350, and >0.350 were used to indicate weak, moderate, and large effect sizes, respectively.^41^ The collinearity between the constructs was assessed by a variance inflation factor (VIF) with values >5 indicative of a probable collinearity issue.

For significant paths, we conducted a mediation analysis to investigate whether pigments mediated the effect of PAHs and turbulence on PSII performance*.* Partial mediation occurs when both the direct and indirect effects are significant, indicating that PSII performance might include effect mechanisms unrelated to the pigment concentrations. The 95% CI for each path coefficient was obtained through bootstrapping (5,000 bootstraps). Both complementary and competitive mediation were considered, to differentiate whether the mediator enhances (complementary) or counteracts (competitive) the direct effect.

## 3 Results

### 3.1 Biological Responses to the Treatments

GLM output indicated that PAHs and sediment resuspension affected several biological responses, while MP did not have any effect (Table 2 and Figures S1–S4 of Supporting Information). Moreover, only the main factors (i.e., PAHs, MPs, turbulence and turbidity) were significant, with no significant interaction effects being detected in any of the models. Except for Chl­(*a+c*), NPQ and antioxidant capacity (ORAC), all responses were negatively (and most severely; *S*_β_ > 0.25) affected by PAHs (Figure S1 of Supporting Information). Although chemical activity had only two exposure levels, it was treated as a continuous predictor. This allowed us to estimate that a 0.01 increase in the chemical activity reduced carotenoids, Fv/Fm and Y­(II) by approximately 10%. It also significantly reduced %C (−4%), %H (−2%), and C/N (−2%). An increase in turbidity by 1 NTU increased NPQ by approximately 20% relative to the average NPQ value. Moreover, this turbidity increase was associated with a reduction in %C (∼1%) and C/N ratio (∼2%), and a decrease in %H (∼0.1%; Figure S2 of Supporting Information). Under high turbulence, significant changes occurred across multiple response variables compared to low turbulence conditions (Figure S3 of Supporting Information). Chl. and carotenoid levels decreased by approximately 2% and 29%, respectively, while NPQ showed a significant reduction of 25%. Elemental composition, including %C, %H, and %N, also decreased significantly, with reductions ranging from 7% to 9%. In contrast, ORAC increased significantly by 21% under high turbulence. Power analysis indicated that the available sample size (*n* = 24) was sufficient to detect significant effects of PAHs on carotenoids, Fv/Fm, Y­(II), %H, as well as the effects of turbulence on carotenoids, %C, %H, and %N. However, all other significant effects may be underpowered (*S*_β_ < 0.37 when *n* = 24, Table 2) due to the limited sample size.

**Table 2:** GLM Models (*n* = 24) for the Algal Responses (Chl, Carotenoids, %Fv/Fm, Y­(II), NPQ, ORAC, %C, %H, %N, and C/N) to the Experimental Factors^b^

response variable	predictors	link	estimate	standard error	Wald	*p* value	*S*_β_
Chl (*a+c*)^a^	PAH activity	log	–1.06	0.706	2.250	0.134	
	turbulence (high)		–0.016	0.008	4.077	**0.043**	0.251
	turbidity		0.001	0.007	0.040	0.842	
	MPs (presence)		0.005	0.007	0.460	0.498	
carotenoids^a^	PAH activity	log	–45.588	16.993	7.197	**0.007**	0.428
	turbulence (high)		–0.290	0.109	7.047	**0.007**	0.418
	turbidity		–0.006	0.096	0.004	0.946	
	MPs (presence)		–0.010	0.076	0.017	0.896	
Fv/Fm	PAH activity	log	–39.261	7.202	29.716	**0.000**	0.969
	turbulence (high)		–0.044	0.099	0.197	0.657	
	turbidity		0.014	0.037	0.145	0.704	
	MPs (presence)		0.013	0.08	0.028	0.866	
Y(II)^a^	PAH activity	identity	–35.923	5.821	38.079	**0.000**	0.774
	turbulence (high)		0.078	0.075	1.099	0.295	
	turbidity		–0.008	0.060	0.020	0.890	
	MPs (presence)		0.104	0.061	2.896	0.089	
NPQ	PAH activity	identity	0.000	1.108	0.000	0.999	
	turbulence (high)		–0.042	0.014	8.599	**0.003**	0.266
	turbidity		0.029	0.011	6.700	**0.010**	0.141
	MPs (presence)		–0.016	0.012	1.956	0.162	
ORAC	PAH activity	log	–2.691	6.346	0.180	0.672	
	turbulence (high)		0.192	0.087	4.821	**0.028**	0.253
	turbidity		0.074	0.060	1.497	0.221	
	MPs (presence)		0.135	0.073	3.463	0.063	
%C	PAH activity	identity	–96.372	47.601	4.099	**0.043**	0.234
	turbulence (high)		–2.278	0.562	16.449	**0.000**	0.546
	turbidity		–1.256	0.487	6.652	**0.010**	0.248
	MPs (presence)		0.028	0.488	0.003	0.954	
%H	PAH activity	identity	–8.303	3.286	6.385	**0.012**	0.407
	turbulence (high)		–0.21	0.039	29.218	**0.000**	0.704
	turbidity		–0.092	0.034	7.524	**0.006**	0.425
	MPs (presence)		0.042	0.034	1.560	0.212	
%N	PAH activity	identity	–6.800	7.603	0.800	0.371	
	turbulence (high)		–0.385	0.090	18.423	**0.000**	0.778
	turbidity		–0.147	0.078	3.556	0.059	
	MPs (presence)		0.006	0.078	0.001	0.935	
C/N	PAH activity	identity	–14.099	4.252	10.996	**0.001**	0.326
	turbulence (high)		–0.011	0.05	0.047	0.829	
	turbidity		–0.104	0.043	5.692	**0.017**	0.163
	MPs (presence)		0.001	0.044	0.000	0.990	

a Response variable was Box-Cox transformed.

b For the categorical predictors, the reference category is indicated in parentheses. Significant *p* values are in bold.

### 3.2 PLS-SEM

#### 3.2.1 Measurement Model

Table S5 of Supporting Information shows the factor analysis results, with NPQ, %C, and %N removed due to low factor loadings (<0.5) and high *p* values. For all LVs, Cronbach’s alpha, rho A, and rho C exceeded 0.7, indicating that the constructs achieved internal consistency (Table S5). In all cases, AVE was above 0.5, confirming convergent validity of the constructs (Table S5), and HTMT ratio of less than 0.9 supported the discriminant validity of the model (Table S6 of Supporting Information).

#### 3.2.2 Structural Model

The full model based on the pathways addressing specific hypotheses (Figure 1) is presented in Figure 2a and Table 3. Among all the LVs, multicollinearity was not an issue (VIF < 5). The nonsignificant pathways, including PAHs → antioxidant capacity (H_1E_), sediment resuspension → PAHs (H_2_) and antioxidant capacity → PSII performance (H_3B_), microplastics → PAHs (H_6_) were omitted (Figure 2a and Table 3), retaining only significant pathways in the final model (Figure 2b).

**Figure 2:**
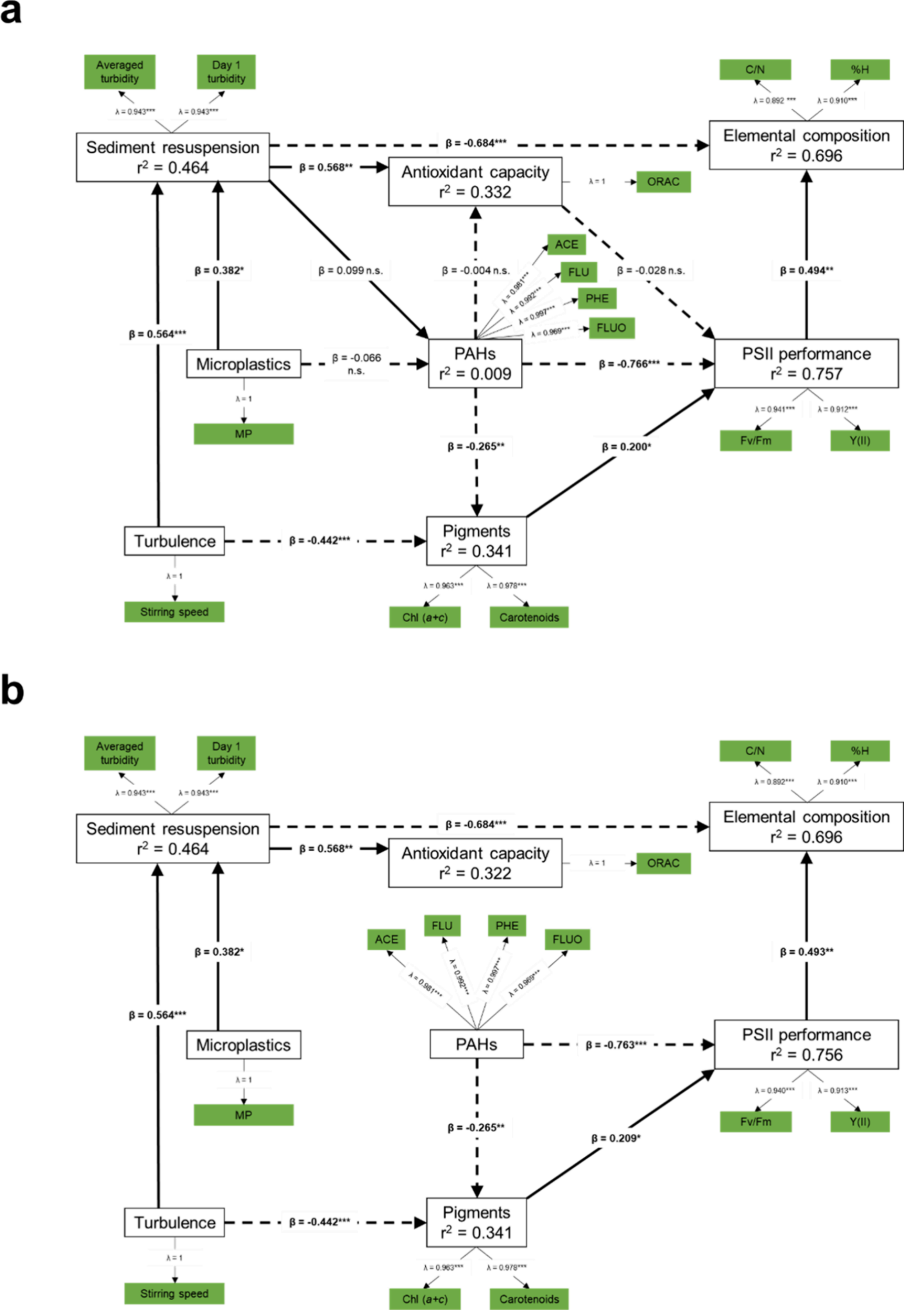
PLS-SEM output (*n* = 24) for the (a) full model with all paths in Figure 1 included and (b) revised model with omitted insignificant paths. Indicators (green boxes), factor loadings (λ), and bootstrapped path coefficients (β) with corresponding statistical significance are shown (n.s. = *p* > 0.05; ** = *p* < 0.01; *** = *p* < 0.001). Indicators with low loadings (<0.5) were removed. Solid line and dashed line indicate positive and negative path coefficients, respectively. Adjusted *r* ^2^ is shown in each endogenous construct.

**Table 3:** Path Coefficients with Corresponding *t* Test Statistics and *p* Values, the Effect Size (*f* ^2^), and Bootstrapped Confidence Intervals (CI, 5 and 95%) of the Full Model (Figure 2a)^a^

hypothesis	path	β	*t*	*p*	*f* ^2^	5% CI	95% CI
H_1A_	PAHs → pigments	–0.265	–2.988	**0.003**	0.097	–0.459	–0.134
H_1B_	pigments → PSII performance	0.200	2.309	**0.015**	0.456	0.028	0.416
H_1C_	PAHs → PSII performance	–0.766	–9.917	**<0.001**	1.987	–0.905	–0.57
H_1D_	PAHs → antioxidant capacity	–0.004	0.530	0.300	0.000	–0.293	0.288
H_1E_	PSII performance → elemental ratios	0.494	3.397	**0.001**	0.787	0.204	0.767
-	turbulence → sediment resuspension	0.564	5.448	**<0.001**	0.593	0.363	0.773
H_2_	sediment resuspension → PAHs	0.099	0.537	0.298	0.000	–0.285	0.632
H_3A_	sediment resuspension → antioxidant capacity	0.568	2.958	**0.004**	0.475	0.127	0.835
-	sediment resuspension → elemental ratios	–0.684	–5.084	**<0.001**	1.530	–0.928	–0.392
H_3B_	antioxidant capacity → PSII performance	–0.028	0.663	0.257	0.000	–0.246	0.211
H_4_	turbulence → pigments	–0.442	–4.100	**<0.001**	0.265	–0.653	–0.245
H_5_	microplastics → sediment resuspension	0.382	2.462	**0.010**	0.273	0.077	0.668

a Significant *p* values for each β are in bold.

The final model had a good fit, with an SRMR value of 0.068 (i.e., below the recommended cutoff values of 0.080) and accounted for 78% of the variance in PSII performance, 68% in elemental ratios, 46% in sediment resuspension*,* 33% in pigments, and 32% in antioxidant capacity (Figure 2b). Bias-corrected *f* ^*2*^ and the bootstrapped CI (Table S7 of Supporting Information) suggested that the observed effects varied from weak (*f* ^2^ < 0.15; PAHs → pigments; turbulence → pigments; pigments → PSII performance; PSII performance → elemental ratios; sediment resuspension → elemental ratios) to strong (*f* ^2^ > 0.35; PAHs → PSII performance; turbulence → sediment ​resuspension; sediment resuspension → ​antioxidant capacity; microplastics → ​sediment resuspension).

As expected, PAHs had significant direct and strong negative effects on PSII performance, whereas pigments had a strong positive effect on PSII performance (Table 3). We also found a weak yet significant negative effect of PAHs on . Moreover, by reducing pigment concentrations, PAHs indirectly affected PSII performance, accounting for 8% of the total effect when considering pigments as a competitive mediator (Table S8 of Supporting Information). Pigments, in turn, had a strong and significant positive effect on elemental ratios*.*

Sediment resuspension was strongly influenced by turbulence (*f* ^2^ = 0.59) and, to a lesser extent, by microplastics (*f* ^2^ = 0.27). In turn, sediment resuspension moderately increased antioxidant capacity and strongly decreased elemental ratios. However, a power analysis indicated that at least 40 samples were needed to adequately estimate the sediment resuspension → antioxidant capacity effect. Turbulence also had a moderate negative effect on pigments, although this pathway also lacked sufficient power, requiring a sample size greater than 27.

## 4 Discussion

The observed algal responses to the combined exposure to PAHs, MPs, and sediment resuspension significantly impacted physiological performance, as indicated by alterations in pigment concentrations, photosynthetic activity, and stoichiometric balance. Of the stress factors tested, PAHs exerted the most pronounced adverse effects across the majority of end points, including a reduction in the photosynthesis performance (H_1A‑C_). Moreover, impairing PSII had downstream effects on carbon and nitrogen stoichiometry, suggesting potential consequences for primary production and food quality for grazers.^43^ The next most influential factor was turbulence and the associated sediment resuspension, which elevated antioxidants (H_3A_) and contributed to the decreased pigments (H_4_) and stoichiometric shifts. The MPs added at the environmentally relevant concentration (∼0.4 mg kg^–1^, which is at the higher end of values reported for coastal sediments),^44^ was the least influential factor for algal physiology, although it facilitated the sediment resuspension as expected (H_5_).

### 4.1 Effects of PAHs

As the primary driver of the adverse effects, PAHs induced phototoxicity through both direct impacts on PSII (H_1C_) and indirectly by reducing pigment levels (H_1B_). In theory, the mixture of the PAH congeners used in this experiment (ACE, PHE, FLU, and FluO) was expected to cause a combination of narcosis, oxidative stress, and phototoxicity, impairing photosynthesis and metabolic processes of the algae. The PSII inhibition was the most profound adverse effect, reducing Fv/Fm and Y­(II) by 42% and 70%, respectively, and accounting for 90% of the total impact. These results support earlier observations that PAHs directly impair the photosynthetic machinery, most likely through sorption into the lipid bilayer of the thylakoid membranes,^45^ which affects the electron transport and membrane protein functioning. As a result, it compromised connectivity between chlorophyll molecules within the light-harvesting complex of PSII,^33,46^ the oxygen-evolving complex and subsequent electron transfer in PSII.^47^ When less energy is passed through the chain, the Y­(II) decrease manifests as reduced photochemical conversion in PSII and low efficiency of photon utilization. The remaining 10% of the PAH effect is attributed to the decline in the pigment content, further reducing energy capture and transport through PSII. As expected, a significant decrease in the carotenoids was found in the algae exposed to PAHs (about 10% drop with 0.01 unit increase in chemical activity) similar to the findings of Kummerová and co-workers.^48^ The most plausible explanation for the pigment decrease is that PAHs inhibit key enzymes involved in pigment synthesis,^49,50^ as supported by transcriptomic data from *Arabidopsis thaliana*, which show downregulation of genes responsible for carotenoid synthesis following PAH exposure.^51^ The reduction of pigments during PAH exposure might also be induced by oxidative stress, as chloroplasts and the plasma membrane appear to be key targets of PAH-induced ROS,^21^ which was one of the rationales for the hypothesized effects (H_1E_). However, we found no significant relationship between the antioxidant capacity in algae and PAHs (thus, rejecting H_1E_), possibly indicating that the antioxidant system performance was sufficient to combat the pro-oxidative processes. Thus, although the reduction in pigments had a strong overall effect on the PSII performance, the direct inhibition of the PSII was likely the main contributor to the decreased photosynthetic efficiency in our study, consistent with the findings of Kreslavski et al.^46^

The observed strong decrease in the elemental ratios (%C, %H and C/N) due to the PSII inhibition (H_1E_) indicate a reduction in the synthesis of organic compounds, reflecting decreased overall metabolic efficiency and nutrient assimilation under PAH exposure. The %C reduction could be attributed to decreased noncyclic photophosphorylation and the activities of key enzymes (RuBisCO and glutamine synthetase), as suggested for mosses.^52^ In plants, the decrease in the C/N ratio, along with the relatively stable %N, can be attributed to impaired synthesis of carbon-rich carbohydrates and lipids, resulting from reduced NADPH and ATP availability under PSII inhibition. This disproportionate impact on carbon compared to nitrogen suggests that in the nutrient-replete media of the experiment, carbon assimilation by photosynthesis and/or increased respiration and maintenance costs were more limiting for growth than nitrogen uptake. Additionally, we observed a decrease in %H in the algal dry mass, likely due to impaired water-splitting capacity, which reduces NADPH production.^33^ As NADPH is the primary hydrogen source for biosynthesis,^53^ a decrease in NADPH limits hydrogen incorporation into organic matter, leading to a measurable decrease in the overall %H content in the algae. In addition, the reduction of organic matter occurs through decomposition, respiration, or photo-oxidation, which break down organic material and release carbon and nitrogen as gases (e.g., CO_2_, NH_3_), thereby reducing their contribution to the remaining biomass.^54^ Thus, the bulk changes in the elemental composition of algal biomass, particularly the C/N ratio, reflect a shift in the balance between photosynthesis and respiration. As PSII efficiency declines, carbon accumulation decreases, lowering the C/N ratio and indicating that respiration and decomposition are surpassing photosynthesis—a response associated with stress adaptation rather than growth.

The four-PAH mixture was dosed at defined chemical activities; because baseline toxicity is additive on the chemical-activity scale, their summed activity these conditions provides the relevant measure of mixture potency, and under such conditions additive effects are expected.^29^ However, it cannot be ruled out that individual PAHs also exert specific phototoxic or pro-oxidant effects, which may lead to synergistic or antagonistic interactions, depending on their chemical properties and biological targets (Table S10 of Supporting Information for possible mechanisms). To further explore potential nonadditive interactions among PAHs, one could compare mixtures with identical total chemical activity but varying congener composition, particularly including or excluding known phototoxic compounds under different light regimes. Experimental designs could also incorporate deviation-from-additivity analyses, such as model deviation ratios, to distinguish between additive and interactive effects. Additionally, extending the PLS-SEM framework to include interaction or moderation terms may help detect whether specific PAHs alter the strength or direction of causal pathways. These approaches would improve our understanding of how individual PAH properties influence mixture toxicity beyond baseline narcosis.

### 4.2 Effects of Sediment Resuspension

Stirring the experimental column introduced two distinct but interacting factors influencing the algae: water turbulence, which enhanced water movement around the algal filaments, and sediment resuspension, which introduced sediment particles into the water column and increased turbidity. While these factors often co-occur in coastal habitats, their relative intensity can vary depending on sediment properties and hydrodynamic conditions: for example, strong turbulence may cause little resuspension in cohesive sediments, whereas minimal disturbance can generate high turbidity in loose or organic-rich substrates. In our study, turbulence contributed to pigment reduction, while turbidity elevated antioxidant capacity and altered algal stoichiometry.

The observed decrease in carotenoid and Chl concentrations under turbulence supports H_4B_ and aligns with earlier reports showing that high turbulence can impair pigment synthesis and stability through oxidative stress and mechanical disruption damaging photosynthetic machinery in algae.^55^ Furthermore, turbulence accelerates the catabolism of growth regulators, such as indole acetic acid and cytokinin, which are essential for photosynthesis and cellular repair.^56^ As turbulence decreased pigment concentrations, it subsequently impacted PSII performance, ultimately leading to changes in elemental composition similar to those induced by PAH exposure.

The interaction between sediment resuspension and algal physiology presents complex outcomes, particularly concerning photosynthetic performance and oxidative status, although the increase in antioxidant capacity (H_3A_) induced by resuspension should be treated with caution due to the limited sample size. Nevertheless, such changes in antioxidant capacity are highly plausible and supported by other studies, where they were attributed to physical damage caused by abrasion and scouring of the algal cell walls, likely resulting in wounding, inducing ROS production (so-called ″oxidative burst″^57^). This response is a common defense mechanism against mechanical injuries in plants and related infections by pathogens. However, contrary to the expected benefits of the elevated antioxidants on photosynthesis (H_3B_), we did not observe an improved PSII performance in concert with these seemingly adaptive responses. A plausible explanation could be that while increased antioxidant capacity reduces oxidative damage, the increased ROS removal may weaken cell-to-cell signaling necessary for photosynthetic adjustment under stress.^58^ Thus, maintaining a balanced redox state within chloroplasts is crucial, as both ROS production and scavenging are needed for optimal photosynthetic regulation.^59^

The decrease in %C, %N, and %H, along with the altered C/N ratio, may be partly explained by mechanical stress from suspended sediment particles. While these changes were largely driven by PAH-induced PSII inhibition, sediment resuspension likely exacerbated the effects through physical abrasion, cellular damage, and the consequent metabolic responses. Such stress may have resulted in the loss of carbon-rich intracellular compounds and increased synthesis of nitrogen-rich stress proteins, as observed in other algae exposed to physical injury.^60^ However, these shifts were secondary compared to those induced by PAHs. Although sediment particle (like silt or clay; Figure S5 of Supporting Information) adherence to algal surfaces could at least partially account for the decrease in %C, %N, and %H, this explanation (albeit relevant) is inconsistent with the observed C/N ratio change. If sediment adhesion was responsible, the increase in inorganic mass would likely cause proportional reductions in both carbon and nitrogen, leaving the C/N ratio largely unaffected. Furthermore, the increase in ORAC levels induced by sediment resuspension provides additional evidence against sediment adherence as the sole cause: if the algal dry mass had increased due to sediment addition, we would expect a dilution effect, leading to a decrease in ORAC levels rather than an increase. These findings suggest that the observed changes in elemental composition and antioxidant capacity result from intrinsic physiological responses to particle-induced stress rather than from mere artifact of sediment adherence.

Sediment resuspension was also hypothesized to increase PAH uptake in algae due to enhanced release from the sediment and uptake by algae (H_2_), but this hypothesis was not supported. The model outcome suggests that sediment resuspension has a weak yet nonsignificant positive effect on the total chemical activity of PAHs in the algae (beta coefficient: 0.099, 95% confidence interval: −0.285 to 0.632). In addition to the nonsignificance, this high confidence interval likely reflects the complexity of PAH mobility in our experiment, possibly influenced by several factors. First, the 7-day exposure period may have been insufficient for PAHs, particularly those with higher hydrophobicity, to equilibrate across sediment, water, and algae. Analysis of the congener-specific concentration measurements in the freely dissolved fraction would help confirm the effects of turbidity on the chemical release from the sediment. Second, the actual bioavailability and uptake by the algae may be influenced by the kinetics of PAHs in the water column containing sediment particles and algae, but also other particular organic matter originating from the sediment and algae, such as bacteria and detritus. Finally, we observed particle deposition on the algal surfaces (Figure S5 of Supporting Information), which could affect PAH values measured in the algae and used for the chemical activity calculations. This particle deposition can lead to overestimation of PAH content, while also potentially affecting PAH uptake. This dual effect complicates the unequivocal interpretation of chemical activity data, as the measured PAHs concentrations may not accurately reflect true bioaccumulation.

The MP addition and, presumably, their longer residence time in the water column did not increase PAH accumulation in the algae; thus, H_6_ was rejected. Given that turbidity itself was not a significant predictor of PAH uptake, the lack of MP effect on PAH uptake is not unexpected. Moreover, MPs only contributed less than 0.001% of the total particulate matter in the system by mass; thus, PAH transfer occurred predominantly via sediment particles. These findings support the current consensus that, while MPs can act as vectors for hydrophobic contaminants like PAHs, their contribution to this transfer in natural systems is minor compared to sediments, due to the overwhelming dominance of sediment particles in mass and surface area available for sorption.^61^

## 5 Conclusions

The combined exposure to an ecologically relevant combination of environmental stressors - PAHs, MPs, and sediment resuspension - altered algal photosynthetic efficiency, antioxidant capacity and stoichiometry. These changes indicated disruptions in photosynthesis, oxidative balance and nutrient acquisition. However, unravelling the sequence of such effects is challenging due to the complexity of stressor interactions, their nonlinear dynamics, and varying physiological response times. By applying advanced statistical methods, such as PLS-SEM, we were able to disentangle direct and indirect pathways, providing insights into the cascade of biological responses and interactions among stressors. These observed effects on algal physiology can potentially lead to decreased primary productivity and nutritional quality, which are fundamental to aquatic food webs and ecosystem functioning.

Prioritising management actions to protect aquatic ecosystems requires a clear understanding of the relative impacts of co-occurring stressors. Our findings identify PAHs as the primary driver of adverse biological effects, acting through multiple pathways and further intensified by sediment resuspension. Current contaminant assessments are largely based on concentration thresholds and do not account for physical stressors or the combined effects of chemical and physical interactions. Therefore, integrated assessment strategies that explicitly address both types of stressors are needed for realistic ecological risk evaluations and effective prioritization of management and remediation efforts.

## Supplementary Material



## Notes

Supplementary experimental details; preparation of PAHs, microplastics, artificial sediment and brackish water; analytical protocols for photosystem II performance, pigments, antioxidant capacity, elemental and PAH determinations; statistical-power calculation; environmental PAH & MP concentration information; PAH uptake data in algae; full experimental-design matrix; model validation for PLS-SEM; description of expected and interactive PAH-mixture effects; five supplementary figures showing stressor impacts and sediment adhesion (PDF)
