# Supplementary material for: Disentangling the Impacts of PAHs, Microplastics, and Sediment Resuspension on Algal Physiology: A Partial Least Squares Structural Equation Modeling Approach
Source: ACS Environ Au. 2025 Jul 23;5(5):490–500. doi: 10.1021/acsenvironau.5c00060 (PMC12447228; doi:10.1021/acsenvironau.5c00060)
Supplement: Supplementary file 1 [file vg5c00060_si_001.pdf]

Supplementary Information

Disentangling the Impacts of PAHs, Microplastics, and Sediment Resuspension on Algal Physiology: A Partial Least Squares Structural Equation Modeling Approach

Hoi Shing Lo[1*], Betty Chaumet[1], Alyssa Azaroff[1], Anna Sobek[1], Sofi Jonsson[1], Elena Gorokhova[1*]

[1]Department of Environmental Science, Stockholm University, SE-106 91 Stockholm, Sweden

*Corresponding Authors:

H.S. Lo (hoishing.lo@aces.su.se)

E. Gorokhova (elena.gorokhova@aces.su.se)

Contents

Text S1. Preparation of PAHs, MPs and artificial sediments 2

Text S2. Preparation of artificial brackish water 3

Text S3. Photosynthesis performance, biomarkers, PAHs and elemental analyses....3

Text S4. Statistical power....5

Table S1. PAHs and MPs concentrations in the environments....6

Table S2. Experimental design and treatments 7

Table S3. PAHs concentrations (μg g^{-1}) in algae. 7

Table S4. LVs and their indicators in the full PLS-SEM model 9

Table S5. Loadings, construct reliability and construct validity 9

Table S6. HTMT ratios 9

Table S7. Bias-corrected f^2 and bootstrapped CI....10

Table S8. Mediation effects based on path coefficients (β) 10

Table S9. Expected effects in aquatic plants exposed to ACE, PHE, FLU, and FluO 10

Table S10. Possible interactive effects in aquatic plants exposed to a mixture of ACE, PHE, FLU, and FluO....10

Figure S1. Effects of PAHs on biological responses....11

Figure S2. Effects of turbidity on biological responses 12

Figure S3. Effects of turbulence on biological responses....13

Figure S4. Effects of MPs on biological responses....14

Figure S5. Fine sediment particles adhere on *C. tenuicorne* due to sediment resuspension. Algae were held by a stainless-steel mesh to avoid dispersion in the exposure column 15

Text S1. Preparation of PAHs, MPs and artificial sediments.

PAHs

We used four PAHs (acenaphthene, fluorene, phenanthrene, and fluoranthene; Sigma-Aldrich, Sigma Chemical Co., St Louis, MO) varying in their hydrophobicity (log Kow: 3.9–5.2) and water solubility (0.1–4 mg L^{-1}) to represent an environmentally relevant PAH mixture of several congeners with a range of chemical behaviour in aquatic environments. PAHs with hydrophobicity exceeding this range were not considered due to their slow release from the sediments which exceeds the timeframe of this study. Stock solutions were prepared in acetone to a final concentration of 8.3 mg mL^{-1} for each PAH by dissolving crystals in the solvent.

MPs

MPs used in this study were generated using films of low-density polyethylene (PE; catalogue number FT5230, Borealis, https://www.borealisgroup.com/products/product-catalogue/ft5230), a polymer commonly found in aquatic environments due to its prevalent use in packaging and disposable products. The PE films were produced by the Research Institutes of Sweden (RISE) and cut into 1 x 1 mm pieces; subsequently mixed into the sediment at a concentration of 10 mg L^{-1} (equivalent to ~0.4 mg kg^{-1} of sediment in the exposure system) for treatments involving MPs. This level of MP load is environmentally relevant to coastal environments[1].

Artificial sediment

Artificial sediment was prepared to simulate Baltic Sea sediments in terms of grain size distribution and composition, having 75% fine (<63µm) and 25% coarse (>63µm) particles. The artificial sediment was prepared according to the OECD guidelines (OECD, 2004). Peat from the garden market was used as an organic carbon source (1% of the total dry mass). The peat was first freeze-dried, ground and sieved at 250 µm; then mixed in a beaker with artificial brackish water (50% of the total sediment dry weight) and a stir bar. The slurry was soaked for 48 h and the pH was adjusted to 5.5–6.0 using $CaCO_3$. The resulting size distribution and organic carbon (OC; 1% among all treatments) are listed below.

**Treatment**	Peat (g)	OC (g)	OC (%)	Quartz sand (g)	Quartz sand (%)	Kaolin (g)	Kaolin (%)	Coarse sand (g)	Coarse sand (%)	Total sediment (without peat) (g)	Fine particles (%)	Coarse particles (%)
Control	34	17	1	1156	68	289	17	255	15	1700	75	25
MPs	22	11	1	748	68	187	17	165	15	1100	75	25
PAHs	26	13	1	884	68	221	17	195	15	1300	75	25
MPs + PAHs	22	11	1	748	68	187	17	165	15	1100	75	25

The PAHs stock solution (in acetone) was thoroughly mixed with the sand to a final concentration of 100 mg kg^{-1} and a total chemical activity of 0.14 in the final sediment. The mixture was covered with aluminium foil under the fume hood for 2.5 hours to evaporate the acetone before adding the other sediment components.

Text S2. Preparation of artificial brackish water.

Artificial brackish water was prepared to represent Baltic Sea salinity of 7‰ using MilliQ water and adding NaCl, KCl, $CaCl_2$, $MgCl_2{\cdot}6H_2O$ and $MgSO_4{\cdot}7H_2O$ at 4.84, 0.17, 0.18, 1.12 and 0.73 g L^{-1} respectively. The pH was adjusted to 8 using a few droplets of NaOH. The bottles were autoclaved and stored at 4°C until use.

Text S3. Photosynthesis performance, biomarkers, PAHs and elemental analyses.

PSII performance

PSII performance was probed using a DUAL-PAM-1000 portable chlorophyll fluorometer (Walz, Germany). Following the protocol established[2], samples were dark-adapted at ambient temperature for 30 minutes before measurement. First, the algae were exposed to modulated measuring light (0.6 kHz, PPFD ≤ 0.1 µmol $m^{-2}s^{-1}$, "weak red light") to record the initial fluorescence (Fo). Subsequently, a saturation pulse light (20 kHz, 300 ms pulse of 10000 µmol $m^{-2}s^{-1}$, "white light") was applied to determine maximal fluorescence (Fm). The actinic light (AL, 531 µmol $m^{-2}s^{-1}$) was then used to stimulate normal photosynthesis for several minutes. During this illumination, steady-state fluorescence (Fs) and maximal fluorescence (Fm') were obtained. Finally, the initial fluorescence (Fo') was measured when AL was turned off, and far-red light (FR) was activated. Three physiological parameters representing PSII performance were derived from these measurements: (1) maximum photochemical efficiency of PSII (Fv/Fm), which indicates the maximum efficiency of PSII when all reaction centres are open; (2) non-photochemical quenching coefficient (NPQ) calculated as:

$$Fv/Fm = (Fm - Fo)/Fm \quad \text{eq. (3)}$$

$$NPQ = Fm/Fm' - 1 \quad \text{eq. (4)}$$

which measures heat dissipation and energy loss; and (3) the quantum efficiency of PSII photochemistry [Y(II)] calculated as:

$$Y(II) = (Fm' - Fs)/Fm' \quad \text{eq. (5)}$$

indicating the efficiency of PSII in converting light to chemical energy, providing the rate of electron transport in photosynthesis. Together, these parameters provide insights into the actual photosynthetic performance under experimental conditions and the overall photosynthetic health of algae[3].

Pigment and total antioxidant capacity analyses

Sample preparation

Algae were lyophilized and weighed to the nearest 0.01 mg. The dry mass of *C. tenuicorne* (1.51–2.28 mg) was used for extraction with acetone, following the modified protocol of Vilg et al[4]. Briefly, the algae were suspended in 0.3 mL of Milli-Q water, together with a garnet matrix and ceramic spheres (Lysing Matrix A, MP Biomedicals, U.S.), and homogenized using FastPrep-24 (MP Biomedicals, U.S.) at 6.5 m s^{-1} for eight cycles of 40 sec with 30 sec. intervals of ice-cooling between each cycle. Subsequently, 0.7 mL of acetone were added to achieve a 7:3 (v:v) acetone:water ratio in the homogenate. The samples were then placed on a rotator (Model 3025, GFL) and extracted at 5 rotations per minute (rpm) overnight at room temperature in the dark. Following extraction, the samples were centrifuged at 4500 rpm for 5 min, and the supernatant was transferred to Eppendorf tubes and stored at -20°C until analysis.

Pigments

The algal extracts were thawed in the dark at room temperature. Chlorophyll (Chl *a* and Chl *c*) and carotenoid concentrations were determined using UV-vis spectroscopy (UV-2600, Shimadzu). Optical density readings were taken at 480, 510, 630, and 664 nm wavelengths, using 7:3 acetone:water (v:v) as the blank. The extracts were diluted if the absorbance at any wavelength exceeded 0.9. Pigment concentrations were calculated based on the measured absorbance at corresponding wavelengths (A_{nm}) by equations (6–8) following the methods of Jeffrey and Humphrey (1975) and Parsons et al. (1984), and taking into account the dilution factors. The pigment concentrations were expressed as µg mg^{-1} of algal dry weight.

$$\text{Chl } a = (11.47 \times A_{664}) - (0.40 \times A_{630}) \quad \text{eq. (6)}$$

$$\text{Chl } c = (24.36 \times A_{630}) - (3.73 \times A_{664}) \quad \text{eq. (7)}$$

$$\text{Total carotenoids} = (7.00 \times A_{480}) - (1.49 \times A_{510}) \quad \text{eq. (8)}$$

Total antioxidant capacity

The ORAC assay, according to the modified protocol of Prior et al.[5], utilizes fluorescein as a fluorescent probe (106 nM), 2,2-azobis(2-amidinopropane) dihydrochloride (AAPH; 152.66 mM) as a source of peroxyl radicals, and Trolox (218 µM) as the calibration standard. First, all algal extracts were diluted with phosphate-buffered saline (PBS, 0.1 M, pH 7.2) at a 1:4 sample-to-PBS ratio (v:v). Subsequently, 25 µL of the diluted samples were combined with 30 µL of AAPH and 150 µL of fluorescein in a well of a 96-well plate. After incubating for 5 min at 37°C, kinetic fluorescence readings were performed (excitation at 485 nm, emission at 538 nm, with readings every 2 min for a total of 65 cycles). The area under the curve (AUC) values for the standards were used to calculate the ORAC values for each sample. These ORAC

values were normalized to the dry mass of the algal samples and expressed in Trolox equivalents (µg mg^{-1}).

Elemental analysis

Approximately 0.5 mg of the freeze-dried algae were weighed with an accuracy of 0.001 mg using a microbalance and transferred to tin capsules (5 × 9 mm; Säntis Analytical). The samples were then combusted and separated on a gas chromatography column (PTFE, 2 m length; 6×5 mm diameter). The amounts of carbon (C), nitrogen (N), and hydrogen (H) were determined using a Thermo Scientific FlashSmart Elemental Analyzer. The combustion reactor was in CHNS configuration, using a quartz reactor filled with quartz wool, copper oxide, and electrolytic copper. Elemental ratios were calibrated with organic analytical standard (AOS) BBOT and controlled with the AOS methionine and acetanilide standards (Elemental Microanalysis). Mean recoveries were within the 99% confidence interval, indicating high accuracy. The coefficient of variation (n = 5) for each certified material was <0.5 %, <0.11 % and <0.10% for C, H and N, respectively.

PAH analysis

The algae were dried with anhydrous Na_2SO_4 in 10 mL glass tubes. PAHs were then extracted using an accelerated solvent extractor (Dionex ASE 350, Thermo Scientific, U.S.) with a 1:1 (v:v) acetone: hexane mixture, with the dried algae spiked with internal labelled standards to achieve a final extract concentration of 200 µg L^{-1}. The solvent extraction was conducted at 100°C for three cycles. The resulting extracts were concentrated to about 1 mL using a rotary evaporator. A 200 µL aliquot was collected and transferred to an amber vial containing 10 µL of anthracene-d10 as recovery standards (Sigma-Aldrich). The samples were stored at -20°C prior to the analysis by gas chromatography-mass spectrometry (GC-MS). The PAH concentrations were then normalized by the concentration of lipid.

Text S4. Statistical power.

We used g*power analysis (G*POWER 3.1.9.7) to estimate sufficient sample size (fixed model, single multiple regression) for R^2 reliability. The input required the estimate of effect size f^2 (0.4), with an alpha error probability of 5% and a 1-beta error probability of 0.80. The number of predictors depends on which construct was evaluated.

Table S1. PAHs and MPs concentrations in the environments. Concentrations are presented as dry weight sediment for PAHs and MPs in water unless otherwise stated.

PAHs		
**Locations**	**Concentration**	**Reference**
Jones Creek Delta State, Nigeria	0.32–48 mg kg^{-1}	6
San Francisco Bay, US	4.5–>100 mg kg^{-1}	7
174 Asian rivers	~0.01–~100 mg kg^{-1}	8
Milwaukee Harbor, US	150 mg kg^{-1}	9
Fjord downstream of an Al smelter, British Columbia, Canada	<150 – 10 000 mg kg^{-1}	10
35-station estuary survey, U.S. Atlantic coast	0.1 – 15 000 mg kg^{-1} (wet weight)	11
This study	100 mg kg^{-1} or 0.14 chemical activity	-
MPs*		
**Locations**	**Concentration**	**Reference**
Bohai Sea, China	6.6–59 mg L^{-1}	12
Hai He Estuary, China	62–155 mg L^{-1}	12
Tianjin coastal areas, China	0.83–75 mg L^{-1}	13
Zhangjiang estuary, China	9.7–81 mg L^{-1}	14
Queen Charlotte Sound, British Columbia (BC), Canada	1.7–112 mg L^{-1}	15
Northwest coast of Portugal	0.8–556 mg L^{-1}	16
North coast of Indonesia Surabaya	0.3–19 mg L^{-1}	17
This study	10 mg L^{-1}	-

*transformation from particle count to mass concentration applied[12]

Table S2. Experimental design and treatments. 4 PAHs (acenaphthene, fluorene, phenanthrene, and fluoranthene) are included in this study. For each treatment group, there were 3 replicates.

Treatment		Sediment resuspension	Chemical activity of PAHs	MPs
1	**a**	High	0.1	Absence
	**b**	Low	0.1	Absence
2	**a**	High	0	Presence
	**b**	Low	0	Presence
3	**a**	High	0.1	Presence
	**b**	Low	0.1	Presence
4	**a**	High	0	Absence
	**b**	Low	0	Absence

Table S3. PAHs concentrations (μg g^{-1}) in algae. Each treatment contains 3 replicates.

Treatment	acenaphthene	fluorene	phenanthrene	fluoranthene
1a	214	382	303	242
	943	1071	842	522
	1113	1208	1142	783
1b	60	81	74	39
	763	622	418	184
	207	227	172	78
3a	1202	1264	1361	1179
	699	804	678	679
	517	522	349	201
3b	118	152	155	110
	284	219	149	60
	228	308	298	226

Table S4. LVs and their indicators in the full PLS-SEM model.

Constructs	Indicator	Description
**Experimental factors (exogenous variables):**		
PAHs	ACE<br>FLU<br>PHE<br>FLUO	Individual chemical activities of PAHs in the algae. Due to high multicollinearity (variance inflation factor (VIF > 5), we classified them as a reflective construct instead of formative (Hair and Alamer, 2022).
Turbulence	Stirring speed	Stirring speed settings of low (6 cm s^{-1}) or high (18 cm s^{-1}).
Microplastics	MP	Presence or absence of MPs.
**Environmental parameter induced by the experimental factors (endogenous variable):**		
Sediment resuspension	Day 1 turbidity<br>Day 7 turbidity<br>Averaged turbidity	Measured turbidity from *Turbulence* and *Microplastics*.
**Algal responses (endogenous variables):**		
Antioxidant capacity	ORAC	Measured total antioxidant capacity by ORAC assay.
Pigments	Chl (*a*+*c*)<br>Carotenoids	Pigment concentrations.
PSII performance	Fv/Fm<br>Y(II)<br>NPQ	Fluorescence-based parameters on the state of PSII.
Elemental ratios	C/N<br>%C<br>%N<br>%H	Elemental stoichiometry in algae reflecting their chemical composition and resource allocation.

Table S5. Loadings, construct reliability and construct validity.

Constructs	Indicator	Loading (p-value)	Cronbach's alpha	Rho A	Rho C	AVE
PAHs	ACE	0.981 (<0.001)	0.990	0.990	0.992	0.970
	FLU	0.992 (<0.001)				
	PHE	0.997 (<0.001)				
	FLUO	0.969 (<0.001)				
Sediment resuspension	Day 1 turbidity	0.943 (<0.001)	0.876	0.876	0.941	0.889
	Averaged turbidity	0.943 (<0.001)				
Pigments	Chl (*a*+*c*)	0.963 (<0.001)	0.939	0.991	0.970	0.942
	Carotenoids	0.978 (<0.001)				
PSII performance	Fv/Fm	0.940 (<0.001)	0.837	0.859	0.924	0.859
	Y(II)	0.913 (<0.001)				
Elemental ratios	C/N	0.892 (<0.001)	0.769	0.773	0.896	0.812
	%H	0.910 (<0.001)				

Table S6. HTMT ratios.

	Microplastic	*PAHs*	*Pigments*	*PSII Performance*	*Turbulence*	*Sediment Resuspension*	*Antioxidant capacity*	*Elemental composition*
Microplastic	-	-	-	-	-	-	-	-
PAHs	0.059	-	-	-	-	-	-	-
Pigments	0.179	0.412	-	-	-	-	-	-
PSII Performance	0.161	0.881	0.585	-	-	-	-	-
Turbulence	0.000	0.325	0.533	0.215	-	-	-	-
Sediment Resuspension	0.409	0.080	0.238	0.078	0.603	-	-	-
Antioxidant capacity	0.123	0.642	0.597	0.585	0.836	0.816	-	-
Elemental ratios	0.291	0.039	0.289	0.194	0.466	0.607	0.522	-

Table S7. Bias-corrected f^2 and bootstrapped CI.

Paths	Original f^2	Bias-corrected f^2	Bias	2.5% CI	97.5%CI
PAHs → Pigments	0.072	-0.257	-0.328	0.082	-0.044
Pigments → PSII performance	0.309	-0.324	-0.633	0.712	0.407
PAHs → PSII performance	1.215	0.652	-0.563	-0.636	-0.636
PSII performance → Elemental ratios	0.687	-0.476	-1.164	-0.901	-0.901
Turbulence → Sediment resuspension	0.593	1.182	0.589	0.361	0.361
Sediment resuspension → Antioxidant capacity	0.475	0.544	0.069	-0.322	0.69
Sediment resuspension → Elemental ratios	1.432	-0.698	-2.13	-1.144	-1.144
Turbulence → Pigments	0.243	-0.921	-1.163	-1.657	-1.657
Microplastics → Sediment resuspension	0.273	0.789	0.516	-0.229	0.379

Table S8. Mediation effects based on path coefficients (β) and their statistical significance for specific indirect effects. Significant p values are in bold.

Type of effect	Effect	β	%contribution	t	*p*
Total effect	*PAHs → PSII performance*	-0.818	-	-19.148	**<0.001**
Direct effect	*PAHs → PSII performance*	-0.753	92%	-9.935	**<0.001**
Indirect effect	*PAHs → Pigments → PSII performance*	-0.065	8% (competitive partial mediation)	-1.834	**0.040**

Table S9. Expected effects in aquatic plants exposed to ACE, PHE, FLU, and FluO.

Compound	Baseline toxicity	Specific toxicity
Acetaminophen (ACE)	Minimal narcosis	ROS generation, oxidative stress, glutathione depletion, photosynthetic disruption
Phenanthrene (PHE)	Narcosis via membrane disruption	Limited; mild metabolic effects
Fluoranthene (FLU)	Narcosis via membrane disruption	Photoactivation, ROS generation, genotoxicity, PSII damage
Fluorene (FluO)	Narcosis via membrane disruption	Photoactivation, ROS generation, pigment damage, lipid peroxidation

Table S10. Possible interactive effects in aquatic plants exposed to a mixture of ACE, PHE, FLU, and FluO.

Type of interaction	Congeners involved	Likely mechanisms
Additive	PHE, FLU, FluO	Shared baseline toxicity via lipid membrane disruption.
Synergistic	FLU, FluO (photoactive) + ACE	Amplified ROS generation; ACE depletes antioxidants, increasing PAH toxicity.
	ACE (low concentrations) + PHE/FLU/FluO	ACE may temporarily mitigate PAH toxicity by diverting cellular antioxidant resources (e.g., stimulated antioxidant production).
Antagonistic	PHE/FLU/FluO (light-dependent)	If light intensity fluctuates (e.g., light periodicity in the exposure), the phototoxicity of FLU and FluO may decrease, potentially unmasking the less photoreactive PHE effects.

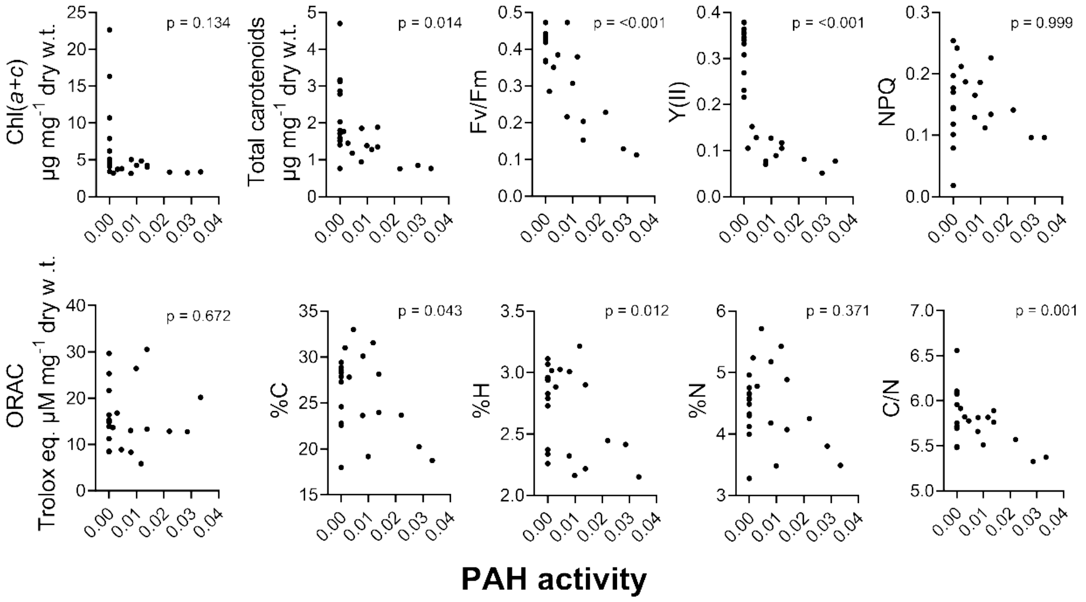

Figure S1. Effects of PAHs on biological responses (n = 24). p-values <0.05 indicate slopes significantly differ from 0 based on the univariate GLMs.

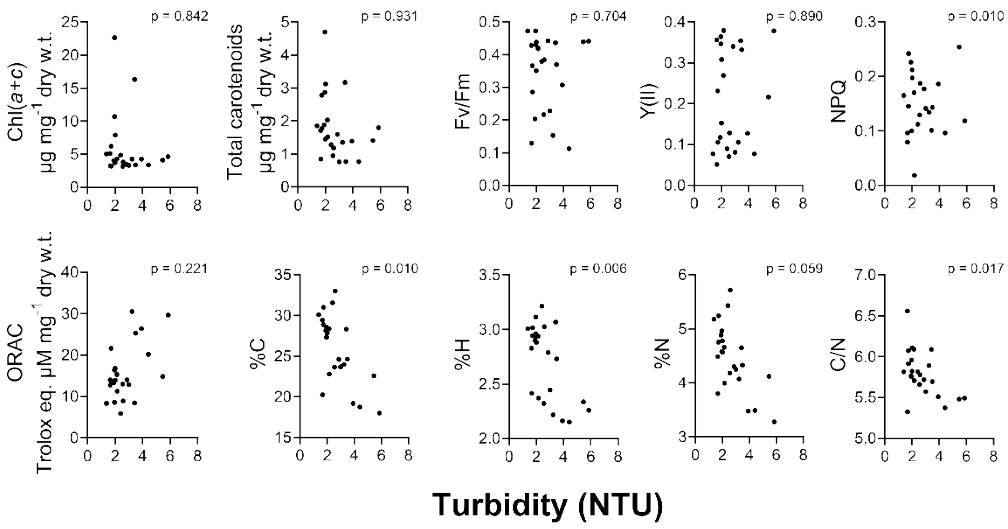

Figure S2. Effects of turbidity on biological responses (n = 24). p-values <0.05 indicate slopes significantly differ from 0 based on the univariate GLMs.

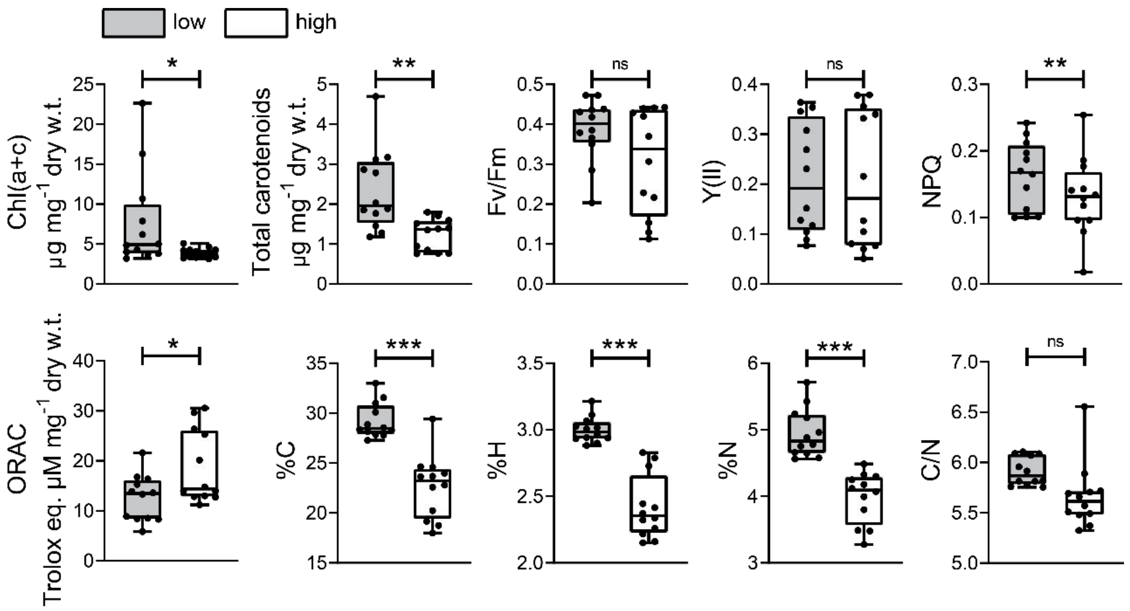

Figure S3. Effects of turbulence (two levels: low and high) on the biological responses (n = 12 for each group). Box-and-whiskers show mean, 25 and 75% percentiles, min and max values. Asterisks (*p < 0.05, **p<0.01, ***p<0.01; n.s. stands for not significant) indicate a statistically significant difference between the groups based on the univariate GLM.

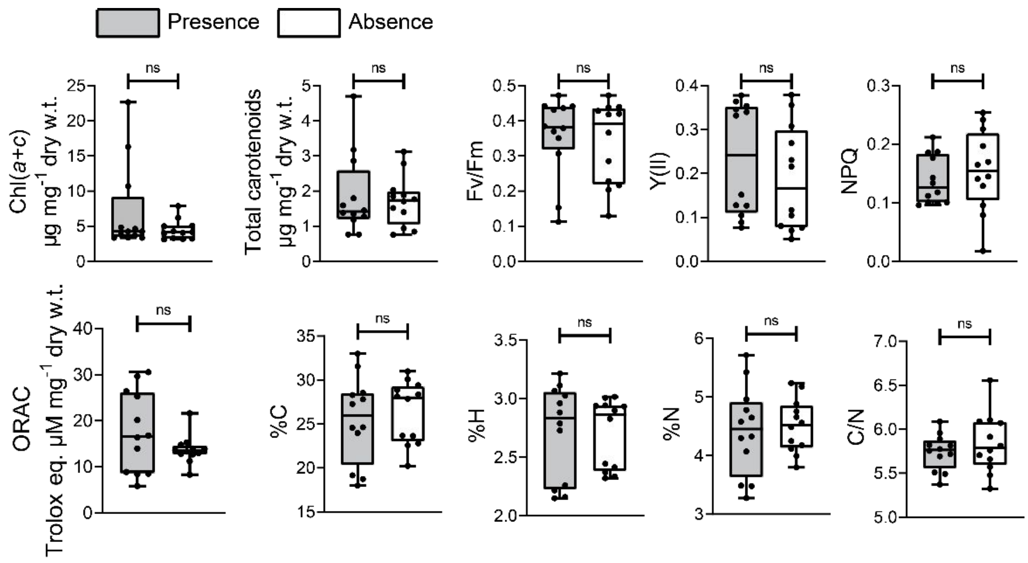

Figure S4. Effects of MPs (two levels: presence and absence) on the biological responses (n = 12 for each group). Box-and-whiskers show mean, 25 and 75% percentiles, min and max values. n.s. indicate no statistical difference between groups based on univariate GLM outcomes.

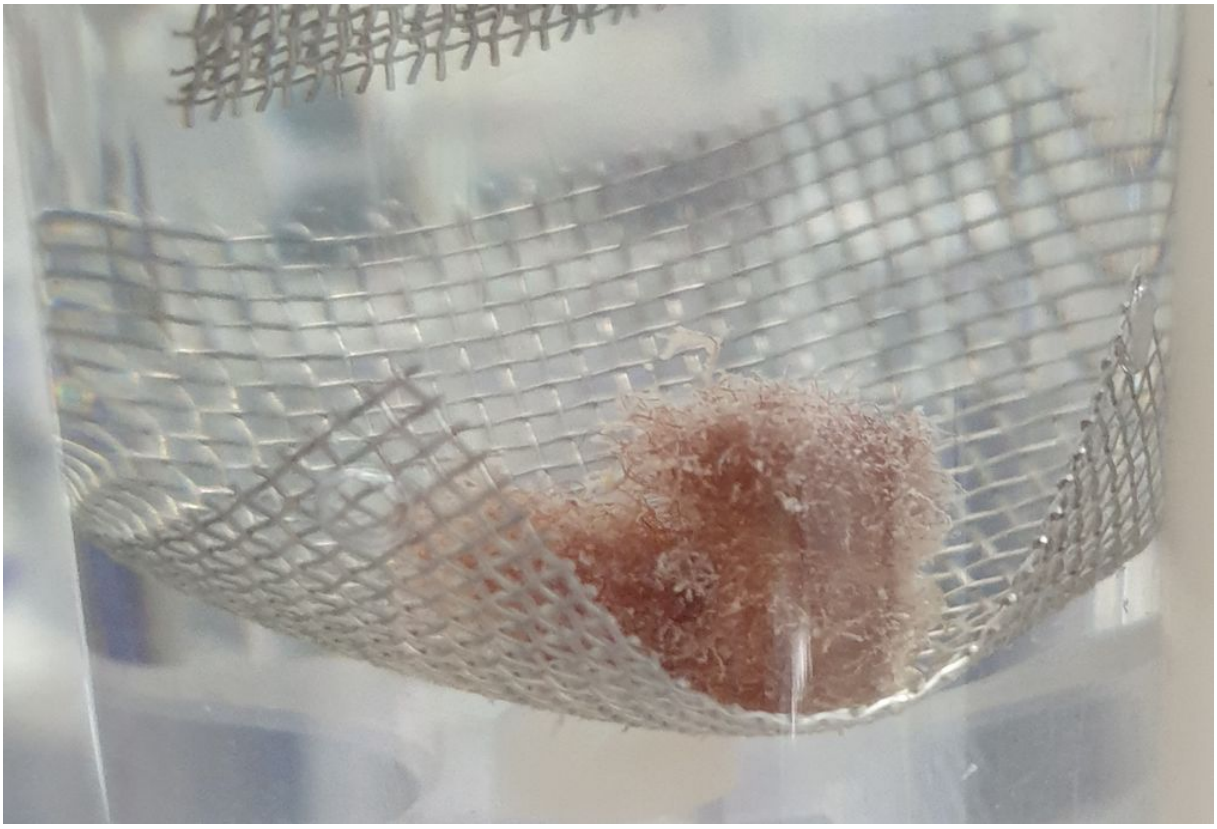

Figure S5. Fine sediment particles adhering on *C. tenuicorne* surfaces under sediment resuspension. Algae were held by a stainless-steel mesh to position them in the exposure system and avoid sinking to the bottom of the vessel.

References

(1) Kye, H.; Kim, J.; Ju, S.; Lee, J.; Lim, C.; Yoon, Y. Microplastics in Water Systems: A Review of Their Impacts on the Environment and Their Potential Hazards. *Heliyon* **2023**, *9* (3), e14359. https://doi.org/10.1016/j.heliyon.2023.e14359.

(2) BURRITT, D. J.; MACKENZIE, S. Antioxidant Metabolism during Acclimation of Begonia × Erythrophylla to High Light Levels. *Ann Bot* **2003**, *91* (7), 783–794. https://doi.org/10.1093/aob/mcg076.

(3) Sánchez-Moreiras, A. M.; Graña, E.; Reigosa, M. J.; Araniti, F. Imaging of Chlorophyll a Fluorescence in Natural Compound-Induced Stress Detection. *Frontiers in Plant Science* **2020**, *11*, 583590. https://doi.org/10.3389/fpls.2020.583590.

(4) Vilg, J. V.; Nylund, G. M.; Werner, T.; Qvirist, L.; Mayers, J. J.; Pavia, H.; Undeland, I.; Albers, E. Seasonal and Spatial Variation in Biochemical Composition of Saccharina Latissima during a Potential Harvesting Season for Western Sweden. *Botanica Marina* **2015**, *58* (6), 435–447. https://doi.org/10.1515/bot-2015-0034.

(5) Prior, R. L.; Hoang, H.; Gu, L.; Wu, X.; Bacchiocca, M.; Howard, L.; Hampsch-Woodill, M.; Huang, D.; Ou, B.; Jacob, R. Assays for Hydrophilic and Lipophilic Antioxidant Capacity (Oxygen Radical Absorbance Capacity (ORACFL)) of Plasma and Other Biological and Food Samples. *Journal of Agricultural and Food Chemistry.* **2003**, *51* (11), 3273–3279. https://doi.org/10.1021/jf0262256.

(6) Ukpebor, J.; Ejeomo, C. PAH Concentrations in Sediment from Jones Creek Delta State, Nigeria: Distribution and Lifetime Cancer Risks. *Tropical Freshwater Biology* **2016**, *25*, 43–56. https://doi.org/10.4314/tfb.v25i1.4.

(7) Jordan, R. E.; Cejas, M. J.; Costa, H. J.; Sauer, T. C.; McWilliams, L. S. PAH Source Differentiation between Historical MGP and Significant Urban Influences for Sediments in San Francisco Bay. *Marine Pollution Bulletin* **2021**, *166*, 112248. https://doi.org/10.1016/j.marpolbul.2021.112248.

(8) Saha, M.; Togo, A.; Mizukawa, K.; Murakami, M.; Takada, H.; Zakaria, M. P.; Chiem, N. H.; Tuyen, B. C.; Prudente, M.; Boonyatumanond, R.; Sarkar, S. K.; Bhattacharya, B.; Mishra, P.; Tana, T. S. Sources of Sedimentary PAHs in Tropical Asian Waters: Differentiation between Pyrogenic and Petrogenic Sources by Alkyl Homolog Abundance. *Marine Pollution Bulletin* **2009**, *58* (2), 189–200. https://doi.org/10.1016/j.marpolbul.2008.04.049.

(9) United States Environmental Protection Agency. Land Treatment of Milwaukee Harbor Sediments Contaminated with PAHs And PCBs. Presented at The Sixth International Symposium on In Situ and On-Site Bioremediation, San Diego, CA, 6/6/2001.

(10) Paine, M. D.; Chapman, P. M.; Allard, P. J.; Murdoch, M. H.; Minifie, D. Limited Bioavailability of Sediment PAH near an Aluminum Smelter: Contamination Does Not Equal Effects. *Environmental Toxicology and Chemistry* **1996**, *15* (11), 2003–2018. https://doi.org/10.1002/etc.5620151119.

(11) Burgess, R. M.; Grossman, S.; Ball, G.; Kady, T.; Sprenger, M.; Nevshehirlian, S. Evaluating Sedimentary PAH Bioavailability Based on Equilibrium Partitioning and Passive Sampling at the Dover Gas Light Superfund Site (Dover, Delaware, USA). *Integrated Environmental Assessment and Management* **2021**, *17* (6), 1215–1228. https://doi.org/10.1002/ieam.4409.

(12) Zhang, J.; Li, Z.; Liang, J.; Liu, G.; Luo, Y.; Zhang, Q. Study on the Mass Concentration Distributions of Marine Microplastics in Estuaries and Coastal Areas. *Water* **2025**, *17* (8), 1136. https://doi.org/10.3390/w17081136.

(13) *Zhu, J. Current status of microplastics pollution in tianjin coastal waters. IOP Conference Series: Earth and Environmental Science* **2020**, *546(3),* 032033.

(14) Pan, Z.; Sun, Y.; Liu, Q.; Lin, C.; Sun, X.; He, Q.; Zhou, K.; Lin, H. Riverine Microplastic Pollution Matters: A Case Study in the Zhangjiang River of Southeastern China. *Marine Pollution Bulletin* **2020**, *159*, 111516. https://doi.org/10.1016/j.marpolbul.2020.111516.

(15) Desforges, J.-P. W.; Galbraith, M.; Dangerfield, N.; Ross, P. S. Widespread Distribution of Microplastics in Subsurface Seawater in the NE Pacific Ocean. *Marine Pollution Bulletin* **2014**, *79* (1), 94–99. https://doi.org/10.1016/j.marpolbul.2013.12.035.

(16) Rodrigues, S. M.; Almeida, C. M. R.; Ramos, S. Microplastics Contamination along the Coastal Waters of NW Portugal. *Case Studies in Chemical and Environmental Engineering* **2020**, *2*, 100056. https://doi.org/10.1016/j.cscee.2020.100056.

(17) Cordova, M. R.; Purwiyanto, A. I. S.; Suteja, Y. Abundance and Characteristics of Microplastics in the Northern Coastal Waters of Surabaya, Indonesia. *Marine Pollution Bulletin* **2019**, *142*, 183–188. https://doi.org/10.1016/j.marpolbul.2019.03.040.
